# An Account of an Epidemic Which Prevailed in Henderson County, Kentucky, during the Winter and Spring of 1845

**Published:** 1845-09

**Authors:** A. J. Morrison

**Affiliations:** Henderson, Kentucky


Art. III.—*An Account of an Epidemic which prevailed in Henderson county, Kentucky, during the Winter and Spring of* 1845. By Dr. A. J. Morrison, of Henderson, Ky.

Before entering upon the details of this epidemic I deem it advisable to refer briefly to a complaint which prevailed with considerable mortality in the same region of country two years previously, called by the people black-tongue, or winter fever. It made its appearance in January, the first subject being an Irishman of intemperate habits, who was seized with a chill lasting ten hours, attended with furious delirium. Swelling of the parotid and sub-maxillary glands came on in a few hours, and was communicated to the face, closing up the eyes so as completely to prevent vision. On the third day after the attack he became comatose, and died the night succeeding. The disease from the appearance of this case became general in the neighborhood, and few families within certain limits escaped it. In every instance it was ushered in with a chill, which not unfrequently lasted fifteen or twenty hours, the surface the meantime being as warm as usual, and often bathed in a profuse perspiration. Perversion of the senses was an unfailing concomitant, especially of the sense of touch, insomuch that when a patient was examined he complained of a piercing pain from the contact of the hand. A uniform complaint was of a sense of fatigue, the patients expressing an earnest wish to be left quiet. In all cases, likewise, mental derangement attended, being sometimes mild, but occasionally of the raving character. Mr. P., a boatman, walked his room but five minutes before he expired, giving orders to his crew. One of his negro women, attacked before him, having been given up as incurable recovered finally, but was idiotic for some time after her recovery. I have seen an intelligent young woman very often since she regained her health, who suffered with the disease in an aggravated form, and for some weeks subsequently discovered a great vacuity of intellect. This affection of the intellectual faculties was frequently preceded by severe pain in the extremities, and this seems to have been one of the most formidable of all the symptoms, as it indicated the commencement of fatal congestion of the great nervous centres. Tumefaction of the neck and face was of very common occurrence, and this often ended in the formation of pus. The tongue for the most part became coated with a black deposit, and this circumstance gave the endemic the name of "*black-tongue.*" The complaint prevailed from January until May, when it disappeared. The number of victims, in all, was 64, being 17 per cent. of the population of the neighborhood visited by the malady. It is remarkable that during the existence of so much sickness in this locality, the country above and below it was healthy.

I am able to give the main features of one of the last cases of this endemic: Mrs. C., a young married woman, nursing a child nine months old, was attacked early one morning towards April with a chill, which continued several hours, and was succeeded by pain in the head, and swelling of the neck and face. The treatment was steaming, stimulants, &c., and at 10 o'clock of the night following she died in convulsions.

The health of this neighborhood continued to be good after the subsidence of the endemic up to the time when the complaint broke out some account of which I propose to give in this paper. The first case that occurred under my observation presented the following features:

Mr. B. was attacked on the 10th of December with a chill under which he labored for the most of the night. Pain in the head followed, particularly of the left side; pulse slow, small and compressible. The treatment consisted in venesection, tartar emetic, to nausea, and purging by calomel and jalap. Evening of the same.—I find him with bowels moved, but pain of the head unabated. Blister to the side, which was the seat of severe pain.

11th. Pain in the side less severe, but that of the head as bad as ever. Patient is dull, sleeps continually, and when roused, begs not to be disturbed. Pulse still slow and easily compressed. Blister to the nape of the neck, and a pill of calomel and ipecacuanha, the latter to be taken every two hours during the day.

Evening. Complains of no pain; is weak and stupid. Bowels moved twice.

12th. Patient still stupid, and without appearance of any special pain. Bowels to be kept open by compound extract of colocynth, blisters to be dressed with stimulating ointment, warm pediluvia, &c. Under this treatment he convalesced slowly, and finally got well.

On the 19th of December, I was called to see Mr. M. who had been seized on the 17th with a chill lasting several hours. The succeeding fever was slight, but the patient complained of severe pain in the right side, and in the forehead. Dizziness, also attended, with slow, soft pulse, and coated tongue. The treatment was bleeding, an emetic, and purgative of calomel and jalap, with blister to the side.

20th. This morning patient is relieved of the pain in his side, but still experiences pain in the head, and has swelling of the glands about the neck. Blister to the nape of the neck, poultices to the swelling, and camphor to be taken every two hours.

In the evening he complained of no pain, but was extremely exhausted, and indifferent to all surrounding objects. No material change was made in the treatment; the patient recovered slowly, but without relapse.

Case III.—In this case as in those just cited, a chill of several hours ushered in the attack. Mr. S., the subject of it, had been ill several days before I was called on the 21st of December to visit him. Pain in the head and right side, skin dry, tongue coated, pulse slow and soft, stupor: these were the characteristic symptoms when I first saw him. A blister was applied over the pain in the chest, and sinapisms to his feet, tartar emetic as a nauseant was administered, and he was directed to take a pill composed of calomel, ipecacuan and camphor at intervals during the night.

22d. This morning the pulse is soft, pain of the side relieved, two motions from his bowels during the night, some irritability of stomach. Discontinue the pills, and prescribe a little brandy and water.

23d. Complains only of weakness. Bowels kept open by a pill of equal parts of rhubarb, aloes, and jalap. Convalescence slow but uninterrupted.

The next case I saw had a different termination. The patient was not treated by me, but I am at liberty to relate the history of his case.

Case IV.—This individual had acted as nurse to the man whose case I have just related. While I was in attendance on that patient he mentioned to me that he had a few days before had an attack of erysipelatous inflammation of the neck extending to the face, that his physician had very promptly checked it, but that he then felt a pain in his right side, for which he spoke of applying a blister.

In a week from this time he had a chill which continued about twenty hours; he was treated by two intelligent physicians, and on the third day, by their invitation, I saw him, when his situation was as follows: He was bathed in perspiration, though his skin was hot, pulse intermitting, subsultus tendinum, picking of the bed-clothes; he died in about twelve hours afterwards, and in a little more than three days from the date of his attack.

I saw no case of the complaint during the next month. Early in February I was called to the following:

Case V. Mrs. H., of good general health, was attacked early in the month, but I was not invited to take charge of her case for several days, a medical man in her neighborhood having been first called to attend her. I found her laboring under pain in the head and side, with cough and free expectoration, pulse 90 and hard, tongue furred. The treatment was bleeding, an emetic, and a purgative, with nauseating doses of tartar emetic, and an epispastic to the region of the chest where the pain was felt.

In the evening all the symptoms were moderated in violence. Ipecacuanha, liquorice, and gum arabic as an expectorant.

4th. Pain in the head still severe, and the patient is dull and sleepy; starts in her sleep. Give a pill of calomel, ipecac., and camphor every two hours.

No better in the evening; bowels have been acted upon; cerebral symptoms still threatening. A blister to the neck, and whole spinal tract to be vesicated with croton oil; pills to be continued.

5th. After the blister drew her mind became composed, and the patient slept well. This morning complains only of weakness.

The subsequent treatment consisted of aperients, wine, camphor and carbonate of iron, and under this her catamenia appeared at the regular period, and she went on steadly improving. Her health is now good.

Case VI. February 11th, I was called in the night to see Mr. K., who had been ill four days; had been bled and blistered, and taken some medicine by the advice of a physician. He complains of a severe pain in his chest: pulse 95, full and strong; bowels discharging a thin yellow mucus, skin bedewed with cold perspiration. I applied a blister to the chest, gave nauseants, and a pill of calomel and rhubarb to be repeated every three hours.

12th. Patient no better. Slept none during the night; pulse still bounding; complains of acute pain in the head. Pills to be continued. In the course of the day they acted, the discharges from his bowels becoming more healthy; the pain in the chest has in a great measure subsided, but the cephalalgia is still severe, and is now accompanied by swelling of the neck and jaws.

13th. He is still restless; pain in the head no better. Applied a blister to the nape of the neck, and vesicated the whole course of the spine with croton oil.

14th. Appears better this morning, having passed a pleasant night; pain of the head not complained of. In the course of the day, however, he seemed to grow suddenly worse; pulse intermittent; extremities cold; fur on the tongue black, and other typhoid symptoms. His case proved a very protracted one, but finally resulted in recovery.

The next four cases in my practice occurred in the same family and will be described in connexion.

The subjects of three of them were men, the other was a boy. In all the disease came on with an enduring chill, which was followed by the train of symptoms already repeatedly described, among which swelling of the cervical glands was uniformly present. The reatment consisted of the remdies used in the cases before given, and the result was favorable in every instance. The enlarged glands were reduced without the formation of pus in any case. At the same house a woman died of the complaint in the care of another physician. Her death happened suddenly, and when she was not thought to be in danger by her medical attendant; she died in convulsions; but concerning the history and treatment of her case I have no certain information.

Case X. February 22d, I was called to see Mr. T., who was attacked with a chill on the night of the 19th, which after prevailing for several hours was followed by a slight fever, pain in the left side of his head, and in the right side of his chest. He had taken no medicine previous to my visit, and I found him in comparative stupor, with costive bowels, weak pulse, and furred tongue. He was bled, and took an emetic of ipecacuan, to which a cathartic succeeded. The remedies afforded very prompt relief, but he continued to sleep almost incessantly through the day.

23d. Pulse this morning slow and feeble; bowels have been acted upon twice; pain of the head persists, and with it there is now swelling of the neck and right jaw. A blister to the nape of the neck; poultices to the swelling; a pill every three hours composed of calomel, ipecac., and camphor, and at night a blister to the side.

24th. He is discharging pus from his right ear, and his head has become easy. Bowels to be kept open by mild aperients—he recovered.

Case XI. I was called, Feb. 16th, to see a negro man, aged 45 years. Twenty-four hours before I saw him he had a chill which lasted all night. I found him with a feeble, frequent pulse, difficult breathing, severe pain in the præcordial region aggravated by lying down, insomuch that he sat up in bed leaning forward on a staff.—He had taken no medicine. I took blood to the extent of four ounces, when symptoms of syncope came on, and I was obliged to desist; but so soon as reaction was fully established I repeated the venesection, drawing about six ounces of blood. Tartar emetic and ipecac. in broken doses to be given through the night, a large blister to his side, and pediluvia.

19th. Pulse compressible and weak, severe pain in his side, coated tongue, dry skin; the nauseants acted freely on his bowels. The pills of ipecac., calomel, and camphor to be given during the day at intervals of two hours, the blister to be dressed with stimulating ointment.

20th. Tumefaction appears about his neck and face, and has increased until he is unable to open his eyes. Anodyne liniment to the swelling, and an aperient medicince containing extract of colocynth.

21st. The swelling has begun to subside, pain in the chest gone, except when he moves in bed. Under the use of mild laxatives he recovered slowly.

The next four cases occurred in the same family and at the same period. It would be unprofitable to enter intc details, since the symptoms, treatment, and termination were the same as in those of which I have related the outlines. The day before I was requested to visit this family one of its members, a lad 14 years of age, died of the complaint. He was attacked on Thursday with a severe pain in his left foot, which shifted the next day to the hand, and was attended by drowsiness. His mother gave him calomel the day following, but on Sunday the stupor continued, and with it he had pain in the head and foot. That day he was visited by a physician, who drew blood, soon after which he was seized with convulsions; in the course of the day became comatose, and died without a struggle, as if falling asleep.

Case XII. Feb. 26th. Mr. W. has been sick for several days; has taken calomel and Cooke's pills until his mouth is sore. He complains of intense pain in his head, and has paroxysms of raving delirium; his pulse is weak and easily compressed. I drew blood to incipient syncope, applied a blister to the back of his neck, and vesicated the spine with croton oil. Castor oil was given to carry off the calomel, to be followed by pills of ipecac. and camphor, one every two hours.

27th. Find patient better in every respect; complains of nothing but weakness. Laxative pills were continued, and the patient had a good convalescence.

In the next case suppuration of the left ear occurred; the illness was of short duration; the treatment chiefly cathartics. In the next an unusually severe chill ushered in the complaint; the subject was a female, troubled with dysmenorrhœa. I found her laboring under cough, and drowsiness; she complained of being blind, was still chilly, pulse compressible, tongue coated. Treatment: an emetic of ipecac., to be followed by rhubarb, and a blister to be applied to the nape of the neck. The day following as she was still drowsy I had croton oil rubbed along the spine, and directed her to take compound extract of colocynth; her body was also sponged with nitro-muriatic acid. She recovered without an untoward symptom.

The next five cases which I had to treat were presented by one family; two of them proved fatal, and the particulars of these I will relate. The patients were attacked with chills which lasted, as in most of the other cases, several hours.—Intense pain of the head succeeded; bowels costive, pulse weak, tongue furred. Miss A., aged 19 years, with the foregoing symptoms, was bled on the 13th of March, took an emetic, was put into a hot bath, and blistered on the neck. Pain in the head next day less intense, but patient is stupid, and indifferent to surrounding objects. Give pills of ipecac. and camphor every three hours, and rub croton oil along the spine.

15th. Her catamenia came on during the day, attended by cramps in the lower extremities, and great difficulty of swallowing; the muscles of the back and neck were also contracted spasmodically; pulse intermitting, mind wandering. Tobacco enemas were used, and the spine was vesicated by croton oil.

16th. Patient decidedly tetanic; retention of urine; soporose. A hot bath, with spirits of turpentine, was used, but no benefit was derived from any application, and she died on the morning of the 17th, four days from the date of her attack

The father of this young woman was seized with chill on the night of the 16th after being much fatigued waiting upon her. He was aged 65 years. I found him laboring under most acute pain in the right side—he compared it to the piercing of a sharp instrument. He was bled, took an emetic, then a cathartic, and had an epispastic to his side.

17th. Patient complains of no pain, but sleeps constantly; if roused, says he is tired, and wishes to sleep. Prescribed a pill of camphor and ipecac., to be taken every three hours, in conjunction with the liberal use of brandy. A blister to the back of his neck.

18th. He still complains of nothing, and sleeps incessantly; has had a severe spasm, and abdominal muscles are now rigid; is roused with great difficulty. At 8 o'clock next morning he expired, having lain for many hours in an insensible state.

A negro boy died of the disease without having complained of any pain, or having been regularly treated. His master could not be convinced that he was in any danger, although I pronounced an unfavorable opinion in his case from the beginning.

Case XIII. I was requested to see Mr. P. in consultation with another physician on the 22d of March. He was attacked on the night of the 17th with a chill which did not pass off until morning, and was followed by a slight fever, and pain in his left side. Dr. B. was called to see him on the night of the 21st, and found the patient troubled with pain in the left lung, cough and free expectoration, his pulse being small and frequent. Nauseants were given, and a blister applied to the side. When I arrived he was flighty, pulse small and easily compressed, tongue coated, bowels constipated. Prescription: a mild cathartic, and ipecac. and gum Arabic as an expectorant.

23d. Pain in the chest less severe, mind more composed, and patient expresses himself as better—Continue the prescription of yesterday, with tincture digitalis.

24th. Patient worse; complains of pain in the upper part of the left lung, pulse feeble, tongue dry and covered with a dark fur, skin alternately dry and moist, mind wandering, picking of the bed clothes, subsultus, &c. A blister was applied to the nape of the neck, sinapisms to his feet, with hot pediluvia; brandy as the patient wishes it.

25th. Less pain of head, cough better, skin moist, pulse slow and regular. He took compound extract of colocynth to open his bowels, and nitrate of potash to subdue the febrile reaction which came on in the course of the day.

26th. Complains of pain in the side when he coughs, but otherwise is easy. A laxative pill and brandy.

27th. Patient decidedly convalescent. No change in the prescription. He recovered slowly. My belief is that the digitalis was injurious in this case, and was probably chargeable with some of the alarming symptoms.

April 21st. I was requested to see M. W., a man of intemperate habits, in consultation with another physician. He was seized with a chill, followed by fever and delirium, and and when I saw him he was thought to be laboring under delirium tremens. He died in two hours after I reached his residence. I am not clear that this was a case of the endemic, though I incline to the belief that it was, and that it was not delirium tremens.

April 24th. I was called to see Moses, a colored man, also in consultation. This patient had likewise been intemperate for a long time, and for some days had complained of indisposition, but the day before I saw him was able to drive his team. That night he was taken with a chill, and in a short time became furious. When I saw him he appeared to me to be in *articulo mortis*, and consequently I gave him nothing. He expired in about an hour. This case, like the preceding, I take to be one of this peculiar endemic, modified and exasperated by the habits of the patient. The chill and delirium were characteristic, and the sudden fatality is explained by the worn-out systems of the inebriates.

Mr. L., æt 66 years, was affected with swelling of the neck on the 21st of April, but after applying a blister to the swelling rode about his farm. The next day he had a chill of several hours' duration. A neighboring physician was sent for, who applied a blister to his chest for a pain of which he complained, and gave him a cathartic which produced copious watery discharges. I saw him for the first time on the evening of the 24th, when he was bathed in a cold perspiration, with a weak pulse, jaws and face much swollen. He sits propped up in bed, breathing with difficulty in the recumbent posture. He had taken laudanum to check the drastic action of the purgative; I ordered him to be rubbed with dry, ground mustard, and gave him brandy freely. The blister on his side drew after a few hours, and with this there was manifest improvement of respiration, in so much that he was able to lie down. His skin grew warm, and his whole situation was greatly improved. Prescription—a pill of calomel, ipecac., and camphor every two hours, and cooling poultices to the swelling of the neck.

25th. This morning he is clear of pain in the chest, expectorates easily, skin comfortable, bowels moved, and he rests well lying. A pill of aloes, rhubarb and jalap to be taken occasionally, in order to keep the bowels soluble. He went on well up to the night of the next day, when going into the passage of his house to the pot he became chilled, returned with a chill to his bed, and soon experienced a renewal of the swelling about the throat, which compelled him to sit up in bed for breath.

27th. I saw him in the evening propped up in bed, his skin bathed in a cold perspiration, and in this state he continued until the evening of the next day, when he expired. But for the unfortunate exposure to a cold atmosphere the result of this man's case, in all probability, had been different. He seemed to be relieved of all his urgent symptoms when the chill was induced with its alarming attendants, from which his enfeebled constitution could not recover.

The last case of this complaint which I was called to treat was presented on the 23d of April. Mrs. P. was taken that day with a chill, having been engaged washing in cold water a day or two previously while her menses were flowing. The day subsequent to their suppression, which took place in consequence of the improper work, she was seized with the chill. The chill was followed by slight fever, and pain in the right side, with soreness and swelling of the neck and stiffness of the jaws. She had been bled, and had taken a dose of calomel, but the medicine had not acted. I gave her a dose of jalap and rhubarb, applied a blister to the side over the pain, sinapisms, and hot foot-bath, and prescribed nauseating doses of tartarized antimony.

25th. Pain in the chest not so severe, but the swelling about the neck very painful. Cooling poultices applied to them with great benefit. Bowels have been opened, and at 10 o'clock the catamenial discharge reappeared, with the relief of all her symptoms. Her bowels were kept open by mild laxatives; the tumor of the neck subsided, and she had a prompt recovery.

With this case my experience in the endemic ended. I saw the first case of it in December, and from that time to the last of April, when that of Mrs. P. occured, was more than four months. In all I treated more than forty cases; I have given the history of a number of them in detail, as affording as perfect a view as I could present of the symptoms of the malady, and of the treatment to which I resorted. From many of the cases one would be inclined to conclude that the disease was not a formidable one, but the termination of some others was so suddenly fatal, that it must be conceded to have possessed a character of decided malignancy. My plan of treatment was simple, and the recovery of my patients was as a general rule strikingly rapid. I will not pretend to say that in all such cases the complaint was not mild, and that much of the result depended upon its being so; but it will not be denied that in other instances where it did not seem to wear a threatening aspect at first it became malignant by neglect, and terminated in death. I submit my history to the profession in the hope that something may be found in its details to interest the practitioner.

Henderson, June 19th, 1845.

